# Clinical results of multilayered biomaterials for osteochondral regeneration

**DOI:** 10.1186/s40634-014-0010-0

**Published:** 2014-08-06

**Authors:** Elizaveta Kon, Giuseppe Filardo, Francesco Perdisa, Giulia Venieri, Maurilio Marcacci

**Affiliations:** Nano-Biotechnology Laboratory, Rizzoli Orthopaedic Institute, Via di Barbiano n. 1/10, 40136 Bologna, Italy; Biomechanics Laboratory, Rizzoli Orthopaedic Institute, Via di Barbiano n. 1/10, 40136 Bologna, Italy

## Abstract

Several techniques have been used during the years to treat chondral and osteochondral lesions. Among them, the emerging trend in the field of osteochondral regeneration is to treat the entire osteochondral unit by implanting cell-free scaffolds, which provide a three-dimensional support for the cell growth and may act themselves as stimuli for an “in situ” tissue regeneration. Various multi-layered products have been proposed that mimic both the subchondral bone and the cartilaginous layer. Among these, three have currently been reported in the literature. One has been widely investigated: it is a nanocomposite three-layered collagen-hydroxyapatite scaffold, which is showing promising results clinically and by MRI even at mid-term follow-up. The second is a PLGA-calcium-sulfate bilayer scaffold: however, the literature findings are still controversial and only short-term outcomes of limited case-series have been published. The most recent one is a solid aragonite-based scaffold, which seems to give promising clinical and MRI outcomes, even if the literature is still lacking more in-depth evaluations.

Even though the Literature related to this topic is quickly increasing in number, the clinical evidence it is still limited to some case series, and high-level studies are needed to better demonstrate their real effectiveness.

## Introduction

Different techniques have been proposed over the years for chondral disease, but treating osteochondral disease is still an arduous challenge for the orthopedic surgeon. Among the currently available surgical approaches, bone marrow stimulation procedures aim at favoring the healing process in the lesion site, taking advantage of the migration of stem cells from the subchondral bone with minimal invasiveness and limited costs, even though paired with well-known limitations (Orth et al. 2012; Kon et al. 2011b). The surgical alternative traditionally consists of more aggressive techniques based on autologous or allogenic tissue transplantation that provides an immediate viable tissue in the lesion site (Gomoll et al. 2012a; Filardo et al. 2014c). The complexity of cartilage-bone interface and the differences between cartilage and subchondral bone layers, including both composition and biomechanical properties, particularly impede successful regenerative treatment (Pape et al. 2010; Madry et al. 2010; Orth et al. 2013). However, progress in the field of biomaterials has led to a new strategy for the treatment of such defects of joint articular surface. Based on the rationale of providing a temporary three-dimensional structure for the growth of living cells and guide for tissue formation, various biopolymers assembled in the form of a scaffold have been developed (Kon et al. 2012a). An ideal scaffold should mimic the biology, architecture, and functional properties of the native tissue, thus promoting cell attachment, proliferation, and differentiation. Other key requirements are biocompatibility and biodegradability: the scaffold should support the early phases of tissue formation and then be gradually replaced by the regenerating tissue. Following this rationale, such constructs were first applied in combination with cultured cells (chondrocytes) such as a 3D support for a better tissue regeneration, thus producing good results even at mid-long term follow-up (Filardo et al. 2014b; Brix et al. 2014). Subsequently, this kind of matrix was applied as “one-step” surgical augmentation to marrow-stimulating techniques, by implanting into the defect alone, thus avoiding any cell addition (Anders et al. 2013; Gille et al. 2013), or in combination with mesenchymal stem cells, harvested and seeded during the same surgical procedure (Gobbi et al. 2014).

In fact, from a clinical point of view, an ideal graft should be an off-the-shelf product, thus allowing easy handling and less expensive one-step procedures, and avoiding problems due to cell manipulation and culture.

With this in mind and an increasing awareness of the crucial role of the subchondral bone in the pathogenesis of articular degeneration, new bi-layered constructs have been developed to reproduce the different biological and functional requirements for the growth of both bone and cartilage tissues, with the aim of providing a treatment to the entire osteochondral unit. In fact, the above-mentioned regenerative techniques showed several limitations when applied to primary osteochondral defects (i.e. OCDs) (Filardo et al. 2012a) or articular lesions in a degenerative context, where the subchondral bone is more frequently involved (Filardo et al. 2013c; Filardo et al. 2012b). As previously reported, some scaffolds displayed a potential to act by themselves as stimuli for the differentiation of resident bone marrow stem cells, by inducing an “in situ” tissue regeneration that allows an orderly and durable osteochondral tissue without the need for any cell augmentation (Kon et al. 2012b; O’Shea and Miao 2008; Keeney and Pandit 2009; Lopa and Madry 2014).

Among the many different multi-layered scaffolds specifically developed to reproduce both bone and cartilage either with or without the addition of cells (Shimomura et al. 2014b), only a few acellular ones have currently been investigated with clinical trials (Table 1). The aim of the present review is to illustrate the available literature evidence, by focusing on the clinical results obtained with these osteochondral scaffolds.

**Table 1:** **Specifics of the osteochondral scaffolds**

**Name**	**Trufit CB™**	**Maioregen™**	**Agili-C™**
**Number of layers**	2	3	2
**Bone phase**	Ca-sulfate	70% HA – 30% Coll I	Aragonite
		40% HA – 60% Coll I	
**Cartilage phase**	Hydrophilic polimer (PGA, PLGA, surfactant)	100% Coll I	Hyaluronate-impregnated aragonite
**Plug size**	∅ 5 - 11 mm cylinders	Square 35 x 35 mm (manual sizing)	∅ 6 - 18 mm cylinders
**Plug depth**	Max 18 mm (to be cut)	6 ± 2 mm (swelling)	15 or 20 mm

## Review

### Collagen-hydroxyapatite based three-layer scaffold: MaioRegen™

**Figure 1:**
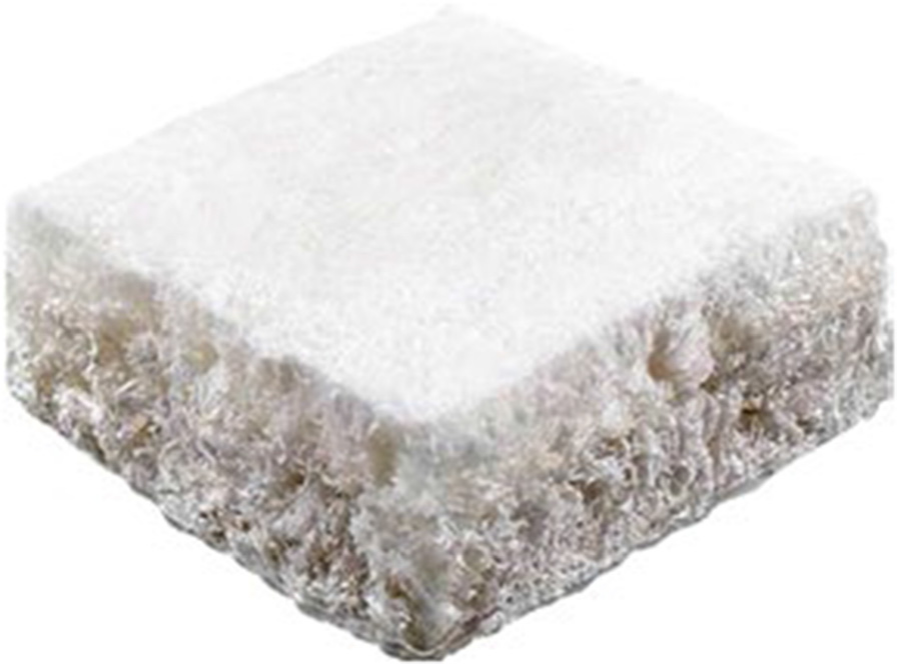
**HA-Collagene type I three-layer scaffold (Maioregen™, Finceramica, Italy).**

The most widely studied multi-layered scaffold is MaioRegen™ (Fin-ceramica, Faenza, Italy), a nanostructured implant consisting of different ratios of collagen (coll) and hydroxyapatite (HA) organized in three-layers (Figure 1; Table 1). The composition of the graft resembles that of the extracellular matrixes of cartilage and bone tissue, and is based on nucleation of HA nanocrystals onto self-assembled coll fibers (Tampieri et al. 2008) to generate a chemically and morphologically-graded biomimetic material. Equine type I coll acts as an organic matrix for the mineralization process, due to its good physicochemical stability, processability, high safety and biocompatibility profile, due to the removal of all potentially immunogenic telopeptides. The mineral phase is represented by magnesium–hydroxyapatite (Mg-HA). Magnesium ions have been introduced to increase the physicochemical, structural, and morphological affinities of the composite with natural bone (Serre et al. 1998). The final design of the scaffold mimics the natural structure of both cartilage and subchondral bone layers: the cartilaginous upper layer is smooth on the surface and consists entirely of Type I coll; the intermediate layer is made of a combination of Type I coll (60%) and Mg-HA (40%); and the lower one consists of a mineralized blend of Type I coll (30%) and Mg-HA (70%). Safety and efficacy of the product have been shown through preclinical studies (Kon et al. 2010a; Kon et al. 2010b), where similar results were obtained with or without loading the implant with cultured autologous chondrocytes. The scaffold appeared to be able to induce an ordered *in situ* regeneration, possibly through progenitor cells from the bone marrow surrounding the implantation site, thus allowing the regeneration of a good quality and well-integrated tissue in the animal model. Moreover, both histological and immunohistochemical evaluations showed type II coll in the cartilage region down to the subchondral bone, and the uniform presence of type I coll in the subchondral layer, even if aspects of ongoing remodeling were still present at 6 months (Kon et al. 2010a). These preclinical findings prompted researchers to bring this method to the clinical practice as a “cell-free” technique.

The preliminary results of this biomimetic graft were reported in the first clinical trial in 2010 in 13 patients (15 lesions): a high-resolution magnetic resonance imaging (MRI) evaluation was performed at 5–8 weeks and 6 months after surgery (Kon et al. 2010b), which showed a complete integration of the implant in 13 of 15 lesions at 5-8 weeks of follow-up, whereas a partial detachment was found in 2 patients. The 6-month evaluation made with MOCART (Magnetic Resonance Observation of Cartilage Repair Tissue) score, showed the ongoing maturation process, with good filling of the lesion and integration of the graft. Subsequently, the same group reported the results of a pilot clinical study at 24 months of follow-up (Kon et al. 2011a). Twenty-eight consecutive patients (9 women, 21 men; mean age, 29.3 years) treated for knee chondral or osteochondral lesions (size 1.5 - 6.0 cm^2^) improved significantly from baseline to the different follow-up times in both the International Knee Documentation Committee (IKDC) and Tegner scores. Further analysis highlighted a faster recovery for patients with higher pre-injury activity level, whereas a slower one was found in patients who presented adverse events, in older ones or those who had undergone previous surgery, and for lesions located at the patella. Finally, most of the MRIs showed complete filling of the cartilage layer and complete integration of the graft. Twenty-seven patients of the same series (Kon et al. 2014a) were then evaluated for up to 5 years’ follow-up, where the clinical results recorded at 24 months were confirmed as stable over time up to the mid-term follow-up. In detail, the IKDC subjective score improved from 40.0 ± 15.0 to 76.5 ± 14.5 and 77.1 ± 18.0, and the Tegner score from 1.6 ± 1.1 to 4.0 ± 1.8 and 4.1 ± 1.9 at 2 and 5 years, respectively. MRI evaluation of 23 lesions was also performed at 2 years and at final follow-up, revealing significant improvements in both mean MOCART score and subchondral bone status over time, although some abnormalities persisted. However, no correlation was found between imaging and clinical outcomes. This biomaterial has also been tested for the treatment of OCD (osteochondritis dissecans) lesions. Twenty-seven consecutive patients affected by symptomatic femoral condyle OCD (average defect size 3.4 ± 2.2 cm^2^) were treated and prospectively evaluated by subjective and objective IKDC and Tegner scores at 2 years’ follow-up. A statistically significant improvement in all clinical scores was present at 1 year, and a further improvement was obtained the following year, with no correlation between size and outcome. The MRI evaluations showed good defect filling and implant integration, although most of the scans presented inhomogeneity of the regenerating tissue and the presence of subchondral bone alterations. However, even in this case, no correlation between the MOCART score and clinical outcome was found (Filardo et al. 2013a). These positive results suggest that an osteochondral approach is key for this pathologic entity, which primarily involves the subchondral bone, and shows good results also for the treatment of large lesions.

Analogously, Delcogliano et al. obtained an encouraging outcome at 24 months’ follow-up on 19 patients affected by large-sized articular defects treated with the same scaffold (Delcogliano et al. 2013), and subsequently demonstrated positive findings in 49 patients of a multicenter study, thus supporting the potential of this product also for the treatment of large osteochondral lesions (Berruto et al. 2014). Similar results were also reported in a bigger series of 79 patients affected by femoral condyles or trochlea lesions (size 3.2 ± 2.0 cm^2^) and evaluated prospectively both clinically and with MRI at 12 and 24 months of follow-up, where a significantly better outcome was recorded in traumatic lesions compared to degenerative ones (Kon et al. 2014b).

Some encouraging findings have been also documented by applying this biomimetic approach in cases of “complex” lesions involving the subchondral bone. A 46-year-old former athlete (Kon et al. 2009) was successfully treated by implanting the scaffold in multifocal osteochondral defects of the medial femoral condyle, trochlea and patella, and concurrently by restoring the correct alignment of a varus knee through a closing-wedge high tibial osteotomy. Good clinical outcome and positive MRI results were recorded at 12 months’ follow-up. Favourable findings were reported also in a 50-year-old woman affected by a tibial plateau osteochondral defect following a Schatzker type II fracture (Filardo et al. 2009). Due to the complexity of the lesion, an integrated mechanical and biological approach was applied to restore the previous anatomic features. Besides filling the bone and cartilage defect with this three-layered implant, the tibial plateau was elevated by an opening-wedge osteotomy and filled with homologous bone graft, and finally a dynamic external distractor was applied to allow early mobilization while protecting the implant. Twelve months later the patient was pain free and returned to a satisfactory activity level, stable until the 4 years’ follow-up. A further case report dealt with an Olympic-level female athlete affected by multifocal degenerative knee lesions. The patient underwent a complex combined treatment: implantation of the scaffold, autologous osteochondral grafting, patellar realignment, and meniscal allograft transplantation to address both joint surface lesions and associated comorbidities. The patient was able to return to high-level competitions within 24 months after surgery (Perdisa et al. 2014). Following these promising outcomes, obtained through a combined mechanical and biological approach as a salvage procedure, a more extensive application was experimented for complex osteochondral lesions. Thirty-three patients affected by “complex” knee lesions, according to defined criteria (Filardo et al. 2013b), were treated by implanting this multi-layered scaffold and combining concurrent procedures to address axial misalignment and meniscal resection sequelae. A good clinical outcome was recorded at 24 months’ follow-up. The clinical results were then compared with those of a homogeneous group of 23 patients previously treated with a similar protocol but with the implantation of a chondral scaffold. A better outcome was found in the osteochondral scaffold group. Finally, the biomimetic scaffold was also tested for the treatment of unicompartmental OA in young patients (Marcacci et al. 2013), to attempt an alternative solution to metal resurfacing. Currently, the main surgical indication in a young and active population affected by unicompartmental OA consists of unloading osteotomies or unicompartmental metal resurfacing (Gomoll et al. 2012b), but the high functional demands and the young age of this kind of patient increase the risk of prosthetic revision. In this challenging population, characterized by a combination of high functional demands and great expectations regarding their recovery on one hand, and a limited choice of treatment options on the other, a solution to delay or even avoid metal resurfacing is highly desirable. The osteochondral scaffold was then implanted in a group of 43 patients affected by unicompartmental OA (Kellegren-Lawrence < =3), with full-thickness focal cartilage lesions in stable knees. Concurrent procedures were performed when required (15 osteotomies, 11 meniscal scaffolds and 9 meniscal allograft implantations). The clinical outcome showed a significant clinical improvement from pre-op to the 3 years’ follow-up. The best benefits were obtained in patients under 40 years old; thus, the authors recommended this surgical approach as a new treatment option for young OA patients.

A recent study tried to define the best fixation method for this scaffold, by performing a comparative mechanical test on a cadaver human knee model (24 scaffolds fixed only by press-fit, 24 scaffolds by fibrin glue) exposed to continuous passive motion (CPM). A statistically significant difference was obtained between press-fit and fibrin glue implants with each score used. Whereas scaffolds implanted only by a press-fit technique are exposed to deformation, delamination, and dislodgement, scaffolds fixed by fibrin glue showed better layer cohesiveness. Furthermore, implant stability and integrity were maintained even when load was applied to simulate early weight bearing. Following these results the authors recommended the use of fibrin glue in the clinical practice, to improve early post-operative stability and integrity of the implant, and thus allowing a safer and faster recovery (Filardo et al. 2014a).

### Poli-lactic (PLGA), poli-glycolic acid (PGA), calcium sulfate biphasic polimer: TruFit™

**Figure 2:**
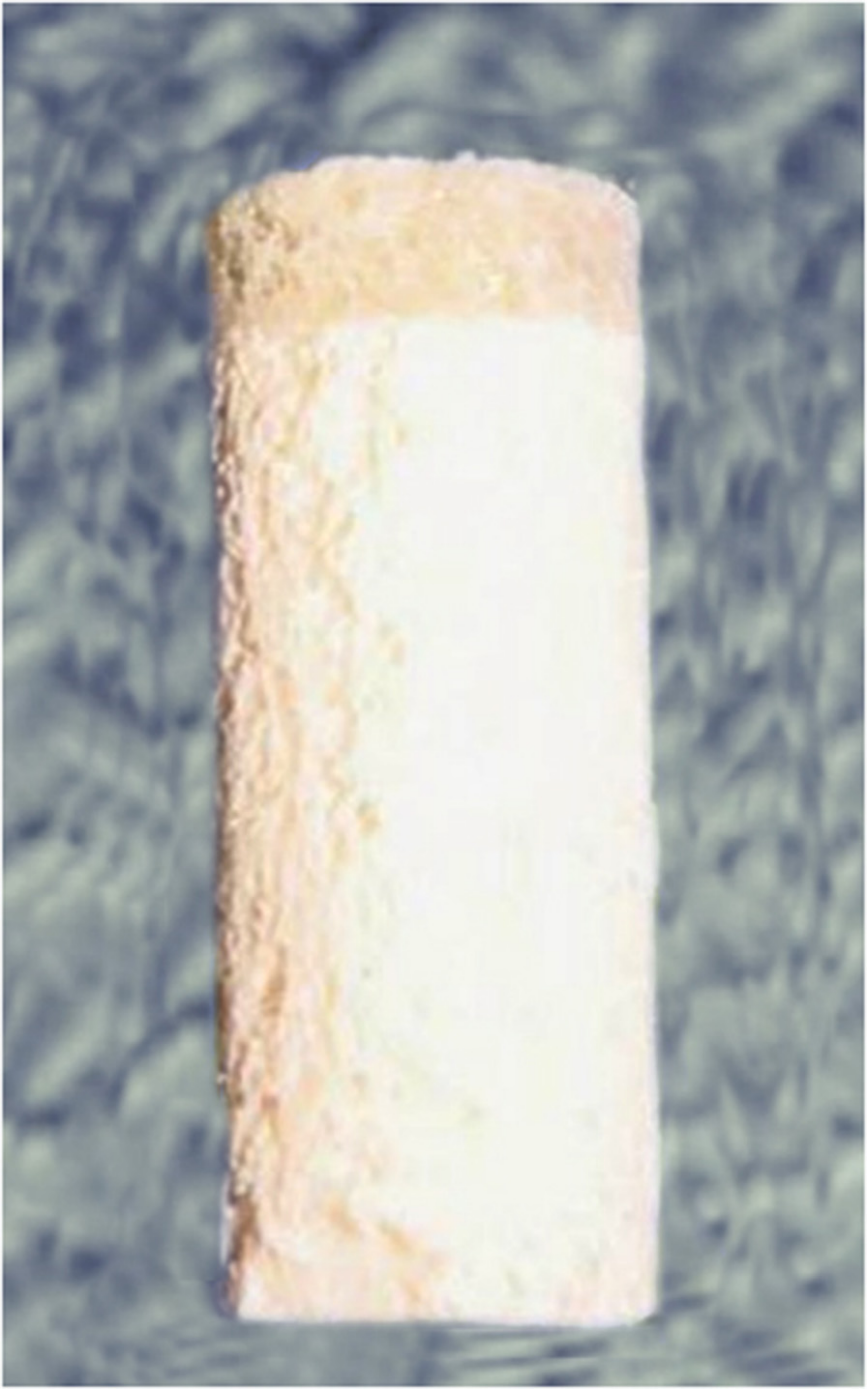
**Polimeric PLGA-PGA and Calciun sulfate bi-layer scaffold (Trufit CB™, Smith & Nephew, USA).**

TruFit™ (Smith & Nephew, Andover, MA) is a bilayer scaffold made of a semiporous 75:25 PLGA-PGA calcium-sulfate biopolymer, shaped into form by different size cylinders (Figure 2; Table 1).

The design should allow a complete resorption over time, which aims to provide a complete filling of the defect with regenerating tissue (Slivka et al. 2001). Promising preclinical results showed an *in vivo* good quality of the regenerating tissue, with similar gross and histological findings either with or without the addition of cells (Niederauer et al. 2000). The scaffold was then introduced into the clinical practice with the indication to backfill autologous grafts donor sites, anyway it has been mainly used off-label, by direct implantation for the treatment of focal articular surface defects, where it showed some controversial results, although long-term durability evaluations are still lacking (Melton et al. 2010; Williams and Gamradt 2008).

The first reports described using this scaffold to fill the donor site during OAT procedures: Bedi et al. (Bedi et al. 2010) found a slow improvement in MRI appearance of the implant site, whereas Barber et al. documented no signs of maturation, osteoconduction, or ossification of the scaffold in any of the 9 patients evaluated with CT scans (Barber and Dockery 2011). Moreover, Quarch et al. (Quarch et al. 2014) showed that filling donor site defects with TruFit™ in patients who underwent OAT, did not produce clinical improvements since the donor site morbidity was already low anyway.

Other studies reported the application of this bone graft substitute for the treatment of articular cartilage knee defects. Also in this case the controversial effectiveness of this implant was shown. Positive findings were documented by Carmont et al. in a case report of a symptomatic 18-year-old patient treated with this scaffold for a large chondral lesion on the lateral femoral condyle. The patient had complete relief of symptoms and was able to return to sports; moreover, the authors suggested that, although an intermediate post-operative interval can be associated with unfavorable MRI findings, the plug appearance may significantly improve at further follow-up (Carmont et al. 2009). With quantitative MRI, using delayed Gadolinium-enhanced technique, Bekkers et al. evaluated the results of 13 patients at a mean of 12 months after the implantation of this plug in their femoral condyles. These findings were positive both in terms of clinical outcome and cartilage-like signal of the graft in its superficial layer (Bekkers et al. 2013).

Conversely, other authors documented poor results. Dhollander et al. (Dhollander et al. 2012) recorded a failure rate of 20% (3 out of 15 patients) at 1-year follow-up and biopsies showing fibrous vascularized repair tissue. An even higher number of failures have been reported when applying this scaffold to patellar defects: 70% of 10 patients treated needed a reoperation due to implant failure within the first 24 months after surgery (Joshi et al. 2012); thus, the authors advised against the use of this scaffold for this kind of lesion. Finally, the comparison of a group of 35 patients treated with this scaffold implantation and 31 patients using mosaicplasty for similar defects, showed significantly higher outcomes for the latter ones (Hindle et al. 2013).

### Crystalline coral aragonite-hyaluronic acid bilayer scaffold: Agili-C™

**Figure 3:**
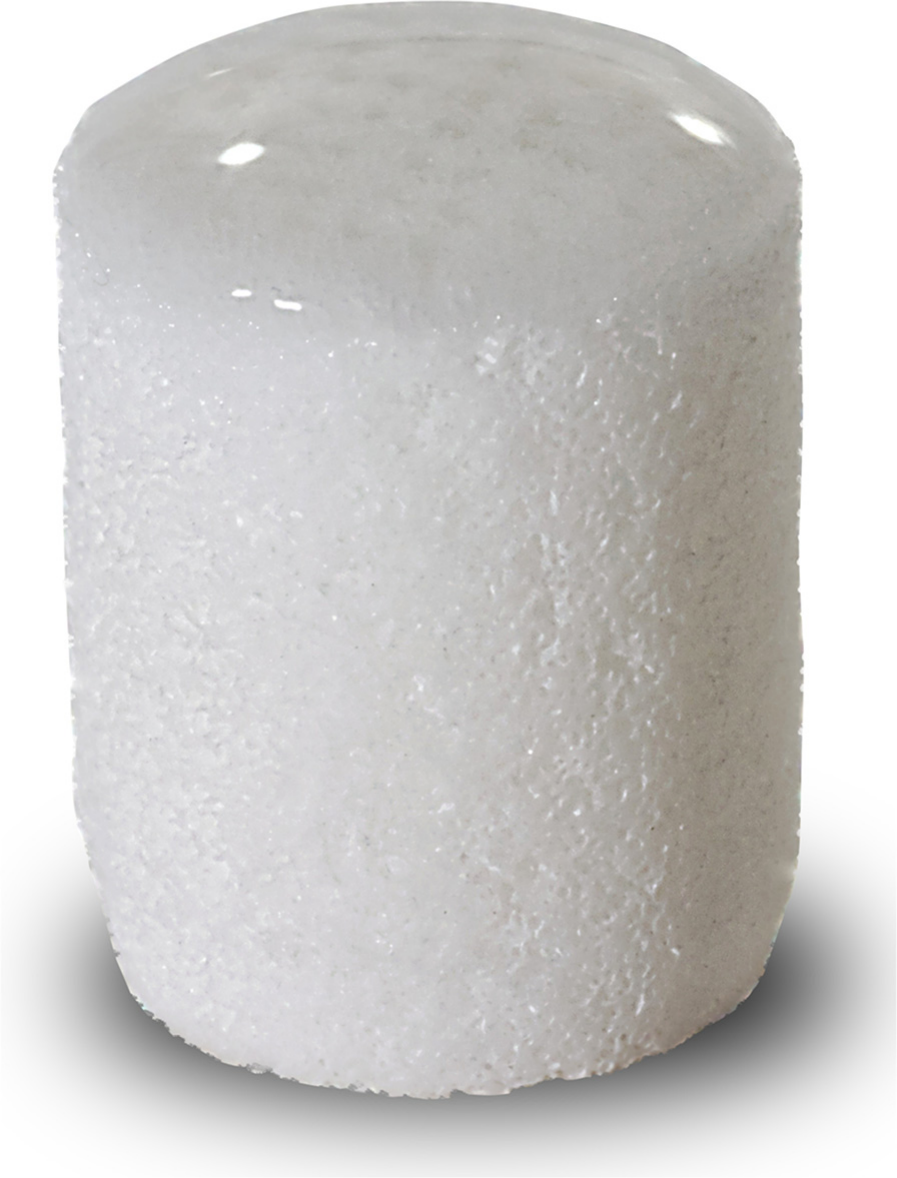
**Aragonite-based osteochondral scaffold (Agili-C™, CartiHeal (2009) Ltd, Israel).**

The most recent literature describes a novel aragonite-based osteochondral scaffold applied into clinical practice (Agili-C™, CartiHeal (2009) Ltd, Israel), developed in shape of cylinders, with a similar surgical rationale than the mosaic-like osteochondral autograft transplantation. It is a rigid cell-free implant consisting of two layers: a bone phase made of calcium carbonate in the aragonite crystalline form, and a superficial cartilage phase composed of modified aragonite and hyaluronic acid (Figure 3; Table 1). Preclinical analysis (Kon et al. 2013b) revealed the safety and potential of this scaffold, thus showing its biodegradability and intrinsic restorative potential. Particularly, its ability to recruit cells from the surrounding tissues, allowed a good regeneration of the entire osteochondral unit to be produced: histological and immunohistochemical stainings showed the presence of collagen type I only in the bone phase and type II in the cartilage layer. This led to the translation of this scaffold as a one-step implantation without any cell augmentation into the clinical setting. Only one case report (Kon et al. 2013a) describing the clinical use of this construct is currently available in the literature: a 47-year-old non-professional sportsman affected by a post-traumatic osteochondral lesion around 2 cm^2^ on the medial femoral condyle was treated successfully and resumed his pre-injury sport activity after 18 months. The MRI evaluation performed at 24 months of follow-up showed promising findings with the restoration of the articular surface.

## Conclusion

The subchondral bone is often involved when the articular surface is damaged and needs to be treated to have a correct and durable restoration of the most superficial layers of the joint. In this context, tissue engineering research has led to the development of several osteochondral substitutes. Even though the association of several biomaterials with different cell strategies is being tested preclinically, these techniques mainly require cell isolation and culture, as well as two different surgical procedures (Zhang et al. 2013b; Shimomura et al. 2014a; Schleicher et al. 2013b; Zhang et al. 2013a; Schleicher et al. 2013a; Duan et al. 2013). This is probably one of the reasons why, despite the favorable results, none of them has already been reported for the clinical use. In fact, the advantages of these new biomaterials include also the possibility to perform a one-step surgical procedure, off-the-shelf availability, a simpler and faster surgical technique, and lower costs. Among commercialized scaffolds, three have been documented in the literature to date. Concerning the polymeric PLGA-calcium-sulfate bilayer scaffold, the reported results are quite controversial, both from clinical and imaging points of view. Conversely, the three-layered scaffold is showing promising results. Also in this case controversial findings have been reported from the imaging point of view, but significant improvements have been repeatedly documented in the mid-term follow-up, with encouraging outcomes even for the treatment of complex osteochondral lesions (including OCDs, large size lesions, unicompartmental OA and combined knee injuries), with the possibility to address also larger articular defects. Thus, this procedure may be a valid treatment option for osteochondral lesions and a possible solution to delay the need for more aggressive approaches in “complex” patients. On the other hand the persistence of an altered signal and a slow maturation process of the osteochondral unit suggest that further improvements are still possible to obtain a better tissue regeneration and an optimal and durable clinical outcome. Finally, a novel aragonite-based product is showing promising preliminary results, but the literature on this new product is still limited and it isn’t still available for the clinical use.

Despite the clinical improvement highlighted by most of the reported studies for a wide range of indications, some common controversial findings seem to emerge with regard to the quality of the regenerative tissue, as testified by alterations in the MRI appearance of the grafts. Experimental studies are ongoing to test new and different multi-layered biomaterials and their interaction with cells and the environment in an in vivo setting. On the other hand further clinical studies are needed to assess the real effectiveness of the available grafts with high level trials, and to better identify the target of patients which would get most benefit from these procedures for osteochondral regeneration.
